# Efficient analysis and extraction of MS/MS result data from Mascot™ result files

**DOI:** 10.1186/1471-2105-6-290

**Published:** 2005-12-07

**Authors:** Florian Grosse-Coosmann, Andreas M Boehm, Albert Sickmann

**Affiliations:** 1 Protein Mass Spectrometry and Functional Proteomics Group, Rudolf-Virchow-Center for Experimental Biomedicine, Universitaet Wuerzburg, Versbacher Strasse 9, D-97078 Wuerzburg, Germany

## Abstract

**Background:**

Mascot™ is a commonly used protein identification program for MS as well as for tandem MS data. When analyzing huge shotgun proteomics datasets with Mascot™'s native tools, limits of computing resources are easily reached. Up to now no application has been available as open source that is capable of converting the full content of Mascot™ result files from the original MIME format into a database-compatible tabular format, allowing direct import into database management systems and efficient handling of huge datasets analyzed by Mascot™.

**Results:**

A program called mres2x is presented, which reads Mascot™ result files, analyzes them and extracts either selected or all information in order to store it in a single file or multiple files in formats which are easier to handle downstream of Mascot™. It generates different output formats. The output of mres2x in tab format is especially designed for direct high-performance import into relational database management systems using native tools of these systems. Having the data available in database management systems allows complex queries and extensive analysis. In addition, the original peak lists can be extracted in DTA format suitable for protein identification using the Sequest™ program, and the Mascot™ files can be split, preserving the original data format. During conversion, several consistency checks are performed. mres2x is designed to provide high throughput processing combined with the possibility to be driven by other computer programs. The source code including supplement material and precompiled binaries is available via  and .

**Conclusion:**

The database upload allows regrouping of the MS/MS results using a database management system and complex analyzing queries using SQL without the need to run new Mascot™ searches when changing grouping parameters.

## Background

For instance, protein identification via MDLC combined with tandem mass spectrometry techniques or other shotgun approaches usually generate huge data sets and compels application of software programs such as Sequest™ [1], Profound [2] or Mascot™ [3]. This produces peptide sequences that need to be grouped in order to obtain protein identifications with several peptides per hit, which increases reliability of the results. Mascot™ groups the peptide results of a single search run automatically. Recombination and merging of search runs is not supported. The data volume limits of Mascot™'s result display tool defined by the underlying computing resource are easily reached and exceeded when applied to a shotgun approach, excluding the opportunity to analyze a huge MDLC experiment at once.

Generally, scientists require their protein identification results in tabular format in order to visualize, filter or sort them by several criteria. Concerning Sequest™, some open source tools for extracting data from its result files already exist, such as Out2Summary from the SASHIMI Project[4] or Sequest Browser™ [1]. For Mascot™, which produces text files in MIME format [[5-10]], such a tool is currently not available as open source. Tools like the ExtParser module integrated in Phenyx [11] convert the preprocessed HTML output of Mascot™'s result display tool rather than the original result file. The parser Mascot2XML of SASHIMI project[4] reads original Mascot™ data and converts into pepXML [12]. This program is available as open source, but does not export all information contained in the Mascot™ file.

For efficient import in spread sheet applications and relational database systems, a straight-forward format is needed, in order to achieve the best performance.

**Figure 1:**
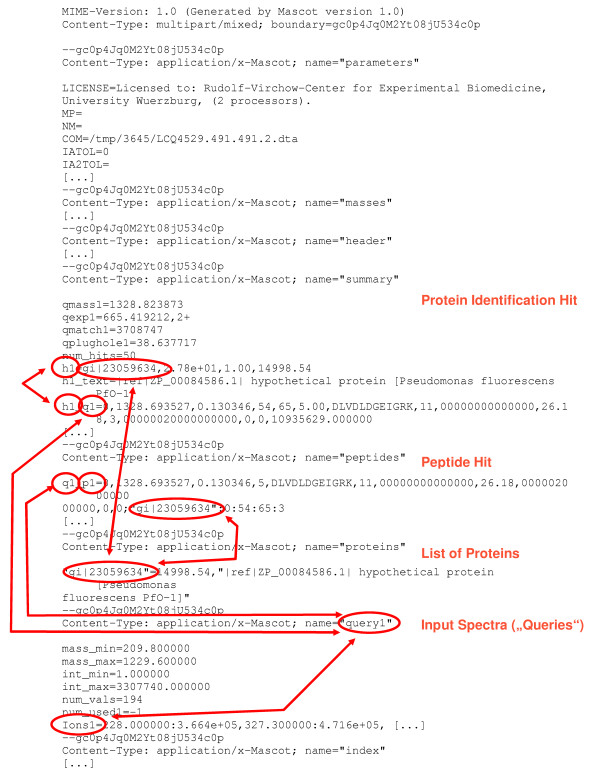
An example of the MIME format of Mascot™ result files is shown in this figure. Wrapped lines are indented. Some lines are removed due to space savings, marked by [...]. The original example file contains 322 lines. Cross-reference links are marked in red.

The MIME format of Mascot™ result files looks as shown in figure 1. Obviously, this format cannot be imported into spread sheet applications or database programs because it contains internal references.

Here, we present the command line tool mres2x that is capable of converting results from original, unprocessed MIME formatted Mascot™ output files (extension .DAT) into a comprehensive tabular format. Extraction of included peak lists into Sequest™'s DTA format is supported, too. Another option allows splitting the original Mascot™ output into several files in Mascot™'s native format according to the number of series of measurements.

An example of running mres2x on Unix/Linux producing tab format output in mascot.tab of the file mascotresult.res stored in /tmp is the following command line:

       ./mres2x -d ./mascot.tab -o tab /tmp/mascotresult.res

## Implementation

mres2x is implemented in C [13], and therefore is portable to several platforms, most notably Windows™ and Unix™. It offers a command line interface. All functionality is controlled by command line parameters that are shown in table 1. A detailed documentation of all command line switches is given in the file Overview.html (see additional file 1) included in the source code package.

**Table 1:** The command line options of mres2x. Parameters for setting the Mascot™'s username, changing line break characters as well as debugging mode exist, too. The usage of mres2x is: mres2x -d destination -o type [-rvfpSuU] filemask_of_input_files, where the last parameter defines the input file(s) including the path and can even be a single file. The input must be in original Mascot™ format, not HTML. The files from the file mask must be in the same directory if the output format is not tab. In case of tab format output, the destination must be a single file, otherwise a folder. mrex2x explicitly expands input file masks. A description of the parameters also can be found in the file Overview.html (see additional file 1) included in the source code package.

**Option**	**Parameters**	**Explanation**
type	s_dta m_dat Tab	Describes the output format. Supported types are: s_dta Sequest™'s dta format. Only spectra data will be exported. m_dat split the input into several output files in Mascot™'s output format, one for each query. Tab write out a tabbed format for direct database upload.
-r		Use CR LF instead of LF as linefeed in data blocks. Some OS need special line feed characters in text files.
-v		Increase verbosity mode by one per occurrence of -v. A maximum of two -v is allowed.
-f		Overwrite files/allow usage of non-empty directories. Usually, the destination directory must be empty.
-p		Preserves files on unsuccessful program termination. Useful for debugging purposes.
-S		Show message indicator even if stderr is a terminal.
-u	name	Set the username to name, if no entry is present and if the tab output format is selected.
-U	name	Set the username to name in all cases if the tab output format is selected. This allows changing the username in the result files of Mascot™.

The program uses one or more Mascot™ result files as input for processing.Its output can be directed to the program's standard output or to a file in case of the tabular output format. Otherwise, an existing directory must be specified as output destination. The converted tabular format is up to 40 percent smaller in size than the original data without any loss of information. It is designed for direct import into relational database management systems, but also can be used with spread sheet applications or other programs for further processing and validation. The tabular format is documented extensively in the file tabformat.html (see additional file 2), where the format of the original Mascot™ result files is implicitly documented, too.

mres2x can be used to split huge Mascot™ result files into single files using the -o s_dat switch, each containing a single query and its corresponding results. This increases performance of reusing the separated results. Typical examples of use are display, analysis or validation by standard tools, such as the bundled result browser of Mascot™. Nevertheless, the main purpose of mres2x is conversion of huge MIME formatted files into a more readable and compact format for efficient direct import into database management systems, using their native import tools.

Several data analysis steps are performed in order to check the validity of Mascot™ files even while processing the input data. Values are checked for their range at this stage. The most detailed validation is performed when producing the tabular format. A full cross-reference check is performed here. Thereby, it is assured that the output is fully consistent. The cross-referenced structure is shown in figure 1.

In case of errors, a cleanup is performed which removes any result files produced so far and the OS is informed by a non-zero return code of the program. It depends on the error whether further analysis of the input file is performed by mres2x. If possible, the algorithm collects errors and prints them out before termination. If included in the input file, Mascot™ warnings are printed to standard error and are available by the calling program.

On success, the message "Operation ended successfully." is written to the standard output. Wrapping programs can easily test for this message or for a return code of zero.

**Figure 2:**
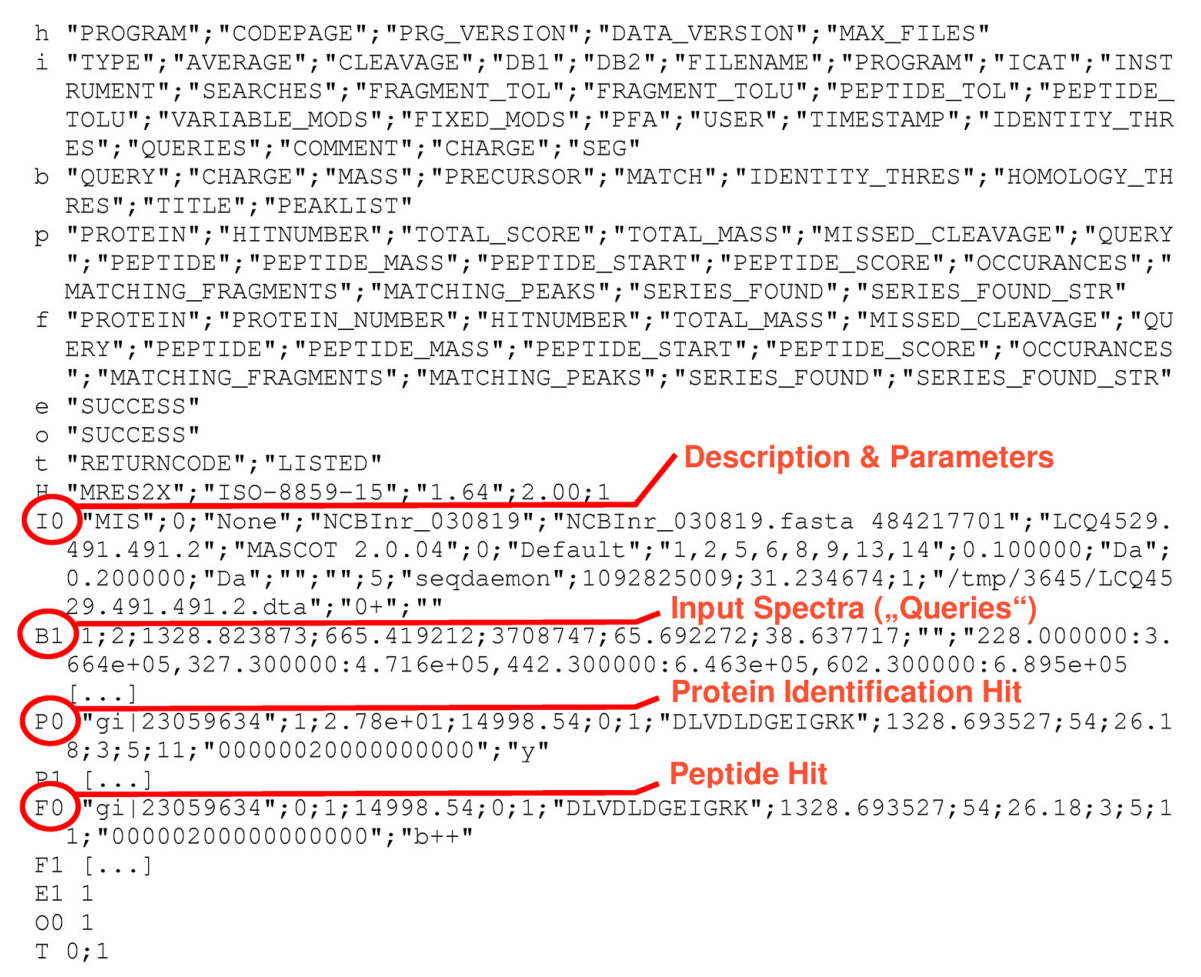
An example of the output of mres2x in tabular format, one record per line. The lines begin with a prefix, indicating the line type. Lower case letters indicate description lines; the corresponding data has upper case letters as prefix which may be directly followed by running numbers. The format is semicolon separated. Chapters are marked and commented in red. The format is described in the file Tabformat.html (see Additional file 2), included in the source code package.

An example of the output in tabular format is displayed in figure 2. The success codes (in this case E1, O0) at the end of a query section (B to E) or a file section (I to O) allow usage of database transaction rollback in case of errors.

mres2x has thoroughly been tested with several thousands of data sets produced by Mascot™ version 2.1.0 and earlier.

## Results

We compared the performance of mres2x with the result viewer named master_results.pl that comes with Mascot™ version 2.0.04, using a 368.74 megabytes large MIME formatted Mascot™ result file, containing 1,565,945 lines obtained from 60,000 MS/MS spectra. Conversion of this file with mres2x lasts 1 minute, 20 seconds, whereas display of this file with master_results.pl using the binary library msparser coming with Mascot™ takes more than 15 minutes on the same computer; the version fully implemented in Perl of master_results.pl would be even slower.

## Conclusion

We introduced a tool capable of converting Mascot™ result files efficiently into other formats, most notable the one designed for direct database import. mres2x is designed to provide high throughput processing combined with extensive error checking and the possibility to be driven by other computer programs. Therefore, mres2x is suitable for integration into computer automated high throughput environments, using direct import into database management systems.

mres2x reads Mascot™ result files and extracts all information in order to store it to another file or files. It currently supports three output formats: First, the original Mascot™ output file can be split into several files with the same format according to the number of series of measurements. Second, the original input peak lists can be extracted into DTA format. Third, a file in tabular format for direct bulk database upload can be created.

In contrast to other formats, such as pepXML [12], protXML [12] and mzIdent [12], mres2x avoids the overhead implied by the need of interpreting the intermediate XML format over again. This allows for importing data directly in a relational database system or spread sheet applications. XML is a storage space consuming format[14] and parsing and interpretation of XML is a time consuming task, decreasing performance of the whole process [15]. Same with other intermediate formats, such as SQT [14]. The tab output format of mres2x is not intended to meet all requirements of the currently discussed file format standardization [[16-19]] and is not designed as a substitute of either XML format mentioned before. mres2x is designed to be used for direct bulk database uploads of Mascot™ results by means of the corresponding database management system, such as SQL*Loader of Oracle™ or bcp of SQL Server™. However, it creates an easy to parse tabular format which makes the creation of translating software to produce other formats nearly trivial. This allows export to any other industry standard.

Storing the results in a database management system allows efficient complex queries on the data such as regrouping of peptide results to protein results without need to research the MS/MS data again and yields time and resource savings as well as increased flexibility.

As the tab output format contains one result record per line, filtering and processing directly after conversion is easily possible, such as for false positives as well as allowing for assembling identifications. The records of protein and peptide results can be distinguished after processing, as the first character of each line indicates the record type.

## Availability and requirements

For compilation a standard C compiler is needed. mres2x can be compiled and run on Windows™ and Unix/Linux. The program is freely available via  and  for download.

## Authors' contributions

FGC implemented the program and made a draft of the manuscript. AB initiated the development. AB and AS contributed with ideas and proofread the manuscript. AB supervised the final testing. All authors have read and approved the final manuscript.

## List of abbreviations used

HTML hypertext markup language

MDLC multidimensional liquid chromatography

MIME multipurpose internet mail extensions

MS mass spectrometry

OS operating system of a computer

SQL structured query language

XML extended markup language

## Supplementary Material

Additional File 1 Documentation of mres2x, this document describes mres2x and how to use it. Click here for file

Additional File 2 Format of the tab output format, this document describes the output of mres2x when tab format is selected. It implicitly documents the format of Mascot™'s result files. Click here for file
