# Supplementary material for: Efficient analysis and extraction of MS/MS result data from Mascot™ result files
Source: BMC Bioinformatics. 2005 Dec 7;6:290. doi: 10.1186/1471-2105-6-290 (PMC1325259; doi:10.1186/1471-2105-6-290)
Supplement: Additional File 1 — Documentation of mres2x, this document describes mres2x and how to use it. [file 1471-2105-6-290-S1.pdf]

Contents

- Goal of the program mres2x
- Parser of the input
- Error handling
- Output format 1: Separated output in Mascot format
- Output format 2: Separated input in Sequest format
- Output format 3: Semicolon separated file
- Destination: Selecting the target files
- Miscellaneous flags

Goal of the program mres2x

This program is designed to give a high throughput in processing with the possibility to be driven by other computer programs.

mres2x reads a Mascot result file and extracts some or all informations and write them to another file or files.

Parser of the input

The parser of the input is implemented using the program *flex*. This is a lexical analyzer program helping one to define patterns and rules for reading data.

The parser should accept even broken files (in content), but the file format needs to be MIME. The parser currently supports various input files and will collect data from each different file. It was a design error to do so, but it is harmless, so it still persists.

The parser is error tolerant in reading and it is very fast. It has some tricks implemented which should reduce memory allocation calls and lookups of variable names.

Error handling

In case of errors a non-null return code of the program is issued. A cleanup is performed which will remove any result files produced so far.

It depends on the error whether some more errors are produced or not. In some cases the algorithms try to collect as many errors as possible.

On success the message *Operation ended successfully.* is written to the standard output.

Output format 1: Separated output in Mascot format

This format can be selected by the command line switch

```
-o m_dat
```

This option creates many output files, one for each query in the original file. The files start with the same base name as the original file added an underscore, a "q" and the query number and a "dat" as the suffix.

In general the data of the original file is copied. Every special informations related to other queries are omitted and the selected query is renamed to the query #1.

There are changes at trivial places only, e.g *queries* in the section *header* is changed.
The number of hits is changed to the maximum detected value.

The proteins itself are modified in that way that the first peptide's hit (*q<num>_p1*) defines the ordner and the content of the section *proteins*.

The hit list of this file represents the best hits of the collection of all queries, yet. In most cases an individual search for just this query would lead to different results for the related proteins. This output format doesn't recompute any value.

Output format 2: Separated input in Sequest format

This format can be selected by the command line switch

```
-o s_dta
```

This option creates many output files, one for each query in the original file. The files start with the same base name as the original file added an underscore, a "q" and the query number, a dot and the charge, and a "dta" as the suffix.

The original mass and the charge is extracted and used as the first line. Subsequent lines contain the measured spectrum.

Note that the order of the queries is changed during a Mascot run.

Output format 3: Semicolon separated file

This format can be selected by the command line switch

```
-o tab
```

All input files are cummulated to one output file. The whole file can be redirected to stdard output which allows pipeline processing.

The format itself is described here.

Destination: Selecting the target files

The target can be selected by the command line switch

```
-d destination
```

destination is a name which should be created or must be empty. This is a directory in case of the output format `s_dta` and `m_dat`. In case of the output format `tab` the destination describes a file name; a hyphen "-" denotes standard output.

The flags change the behaviour of the requirements for *destination*.

Miscellaneous flags

Flags changing the behaviour

<table>
<tr><th>flag</th><th>purpose</th></tr>
<tr><td>-v</td><td>Increases the verbosity level. The maximum verbosity level is 2.</td></tr>
<tr><td>-r</td><td>Uses the pair carriage return/linefeed (CR LF) instead of a plain LF on output. Note that CR LF is used already in MIME header lines to fulfill RFC requirements in the m_dat output format.</td></tr>
<tr><td>-f</td><td>Overwrite existing files or use even non-empty directories.</td></tr>
<tr><td>-p</td><td>Usually files are removed after an insuccessful run. This flag preserves created files from destruction in case of an error.</td></tr>
<tr><td>-S</td><td>Messages of any kind are written to standard error. These messages get an indicator if written to a file or a pipe. With this flag the indicator is used in any case, in particular for terminals.

Used indicator elements
<table>
<tr><th>indicator</th><th>indicates</th></tr>
<tr><td><F></td><td>A fatal error occurred. The complete process dies immediately.</td></tr>
<tr><td><E></td><td>An error occurred during the processing of a file. The processing of this file is stopped, but other input files may be processed if possible.</td></tr>
<tr><td><I></td><td>This is an informational message. The amount depends on the flag -v.</td></tr>
</table></td></tr>
<tr><td>-u name</td><td>This flag is used only with the tab output format. The username is set to name if no username is present or the original name was set to the empty string.

See below.</td></tr>
<tr><td>-U name</td><td>This flag is used only with the tab output format. The username is set to name in every case and overwrites every other name.

See above.</td></tr>
</table>
