# Supplementary material for: Efficient analysis and extraction of MS/MS result data from Mascot™ result files
Source: BMC Bioinformatics. 2005 Dec 7;6:290. doi: 10.1186/1471-2105-6-290 (PMC1325259; doi:10.1186/1471-2105-6-290)
Supplement: Additional File 2 — Format of the tab output format, this document describes the output of mres2x when tab format is selected. It implicitly documents the format of Mascot™'s result files. [file 1471-2105-6-290-S2.pdf]

Format of the `tab` output format

This format is the main goal of the whole program. It is designed to be the pre-step of a bulk data uploader of a database.

- Structure
- Code H, The Header line
- Code T, The Termination line
- Code I, An Input file description line
- Code O, An input file ending line
- Code B, The Beginning of a new query processing
- Code E, The Ending of a query processing
- Code P, A Protein data line
- Code F, A peptide data line

Note that the output contains the default value instead of empty lines or missing lines in the original input files.

Structure

The file is line oriented.

Empty lines may be in the file and should be ignored.

Every other line has a character describing its purpose in column 1. A number may follow immediately. The true content follows after a blank.

Roughly, a line has this format:

```
cnum_data...
```

where **`c`** is a purpose code, ***`num`*** is the optional number and ***`data`*** is the content of the line.

Characters in lower case indicate a description of a line. The corresponding upper case character contains data without a description. E.g. the fictional lines

```
m "ID";"MESSAGE"
M7 42;"This example is rather stupid."
```

show that a line pair exists for the code *M*. The first like with the lower case *M* acts as a pattern how to parse the next line. `M7` indicates that this is the 7th item of *M*. The counting itself is independent of data specific id numbers as shown in this case. *42* is the value of the *ID* entry, *"This example is rather stupid."* is the value of the *MESSAGE* entry.

Some contraints of the structure:

- The format is line-oriented.
- Empty lines have to be ignored. The following contraints do not apply to empty lines.
- The first character defines the line type.
- By convention, a lowercased character acts as a pattern while an uppercased character contains data fitting the pattern.

- A number is following the code immediately in many cases of data lines. This number is for the user's benefit when analyzing errors.
- A blank delimits the code and the content part.
- The content fields are separated by a semicolon.
- A content field may be either a string in double quotes or a number.
- Numbers may be floating point numbers (even in exponential notation) or whole numbers. A whole number may be used to represent a field which usually contains floating point numbers.
- Strings do not contains characters below ASCII-Blank or double quotes. These characters are translated to blanks.
- The content field do not contains other characters. The reader should accept both LF as CRLF as line terminators.

See *Code H, The Header line* for some other important informations.

Code H, The Header line

This line is given once directly after the pattern lines.

Fields of the header line

name	content
**PROGRAM**	Name of the producer. This is **"MRES2X"** in all cases.
**CODEPAGE**	The used codepage while processing the input files. This value may not be of any interest, because the input data was generated in another codepage.
**PRG_VERSION**	This value is a string and represents the version of this program in our source repository. Even small changes should change tis number.
**DATA_VERSION**	This value is a number with two fractional digits. The fractional part is increased if new fields have been added to the various content lines. The integer part is increased on structural changes, e.g. new line codes or removal of fields or lines.
**MAX_FILES**	This value contains the number of input files that are about to be processed. The final number of processed files may not be the number of input files mentioned here, because mres2x does a single step processing and continues its work even when errorneous input files have been detected. The buggy file names are included in this number.

Code T, The Termination line

This line is given once as the last line in the output file. A severe error can be considered if this line doesn't appear. A roleback is suggested for the last data set.

Fields of the termination line

name	content
**RETURNCODE**	Code that will be returned to the caller of the program. This is either 0 or 1 currently. 0 indicates full success, 1 indicates at least one error.

**LISTED**	In opposite to Code H, field MAX_FILES, this entry contains the number of actually listed input files in the output file. Note that even errorneous data sets are counted if they are shown at least partially.

Code I, An Input file description line

This line introduces the processing of a new input file. All following lines up to EOF (in case of en error) or a Code O-line are related to this individual file.

Fields of the input file description line

name	content
**TYPE**	Processed data type. **"MIS"** indicates an MS/MS run. **"PMF"** indicates a peptide mass fingerprint. Other elements are not planned to be supported.
**AVERAGE**	This is the kind of processing that has been made. 0 indicates a monoisotopic computation, 1 a computation with average mass values.<br>See parameters:MASS
**CLEAVAGE**	This is the chemical digest used in this experiment. A typical value is **"Trypsin"**.<br>See parameters:CLE
**DB1**	This is the description of the database used. There is no list or nomenclatura to use, so expect differences where no differences are and vice versa. This name is hopefully comparable with other experiment's **DB1** field. An example is **"yeastsgd"**.<br>See parameters:DB
**DB2**	This is a more exactly description of the database used. There is no list or nomenclatura to use, so expect differences where no differences are and vice versa. An example is **"yeast_all_sgd.fasta 3018992"**. The used filename by Mascot is listed, followed by the number of residues. The intention for this field is to create a possibility to distinguish between several experiments with the same database but with a different dataset, which may result in incomparable values.<br>See header:release and header:residues
**FILENAME**	This field contains the filename of the experiment. The path components are stripped off as well as the suffix (as far as it is well known). This is **not** the input file name. It is the name of the file that has been listed in the input file.<br>See parameters:FILE
**PROGRAM**	This field contains the program name that produced the input file. A typical value is **"MASCOT 2.0.04"**. The text "MASCOT" is constant, the number part is the version string passed in the input file.<br>See header:version
**ICAT**	This field contains either 0 or 1. ICAT has been enabled if 1 is given. ICAT is a dangerous field. It changes the results extremely but is hard to detect if activated incidentally.<br>See parameters:ICAT

INSTRUMENT

This field contains a string describing the used instrument. The big differences between instruments become manifest in the following parameter SEARCHES. Note that `"Default"` is a common value and is it wrong in most cases where you don't use your microwave oven as the instrument.
See parameters:INSTRUMENT

SEARCHES

This field contains a list of numbers. Each number selects a different ion series. The overall selection is done by chosing the instrument, which is translated to this ion series list in the file *fragmentation_rules*. Currently used rules (at RVZ):

1. singly charged
2. doubly charged if CHARGE >= 2
   (not internal or immonium)
3. doubly charged if CHARGE >= 3
   (not internal or immonium)
4. immonium
5. a series
6. a - NH3 if a significant and fragment includes RKNQ
7. a - H2O if a significant and fragment includes STED
8. b series
9. b - NH3 if b significant and fragment includes RKNQ
10. b - H2O if b significant and fragment includes STED
11. c series
12. x series
13. y series
14. y - NH3 if y significant and fragment includes RKNQ
15. y - H2O if y significant and fragment includes STED
16. z series
17. internal yb < 700 Da
18. internal ya < 700 Da
19. y or y++ must be significant
20. y or y++ must be highest scoring series
21. z+1 series
22. d and d' series
23. v series
24. w and w' series

See parameters:RULES

FRAGMENT_TOL

This field contains the fragment mass tolerance value. This is the radius of the window around the measured points that must be hit to let a fragment fulfill its "hit" criteria.
See parameters:ITOL

FRAGMENT_TOLU

This field contains the fragment mass tolerance unit. This is either `"Da"` or `"mmu"`.
See parameters:ITOLU

PEPTIDE_TOL

This field contains the peptide mass tolerance value. This is the radius of the window around the computed peptide masses that must be hit by the precursor mass to let a peptide fulfill its "hit" criteria.
This value has an active influence on the intensity threshold(s), because the count of matching theoretical peptides in the window defines the threshold.

See parameters:TOL

PEPTIDE_TOLU

This field contains the peptide mass tolerance unit. This is either **`"Da"`**, **`"mmu"`**, **`"%"`** or **`"ppm"`**.
See parameters:TOLU

VARIABLE_MODS

This field contains a comma-separated list of modifications. Each modification has this form:

```
special=diff=description
```

or

```
special=diff[neutral]=description
```

`special` is a special character selected by mres2x to be appended to the modificated amino acid character later described. The special character is choosen from the this list `"@~#§!^°:;`'/={}[]()/"` from left to right.
`diff` is the mass difference between the used value and the standard value (*u* - *s*). Note that Mascot uses the last amino acid in the *mod_file* to compute the value. Many things may go wrong if more than one mass difference has been applied to the various residues of one modification.
`[neutral]` is given only if a neutral loss exists. `neutral` is a signed value describing the gain to the modification mass. E.g. `@=79.978699[-97.995200]=Phospho (T)` shows a modification gain of roughly 80 Da, but in case of a neutral loss you will have more or less 80-98 Da, which is an overall loss of 18 Da.
`description` is a freely choosen text by the modifier of *mod_file* hopefully describing the modification enough.
An empty string is possible for this variable.
See masses:delta*i* and masses:NeutralLoss*i*

FIXED_MODS

This field contains a comma-separated list of modifications. Each modification has this form:

```
AA=diff
```

`AA` is a one of the characters used for amino acids, one of the atoms `Hydrogen`, `Carbon`, `Nitrogen`, `Oxygen`, the electron mass `electron` or one of the two terminus placeholders `C_term` or `N_term`.
`diff` is the mass difference between the used value and the standard value (*u* - *s*). Default values are the weight of the molecules H and OH for the N terminus and the C terminus.
An empty string is possible for this variable.
See the section masses

PFA

This field contains either a whole number >= 0 which is the partials factor. This is the maximum number of missed cleavages Mascot will compute with. The default value is 0 despite the documentation.
See parameters:PFA

USER

This field contains the user name associated with the experiment. Note that mres2x has the opportunity to overwrite this field.
An empty string is possible for this variable.

	See parameters:USER and the flags -u and -U
**TIMESTAMP**	This field contains the unix time stamp of the run of the analyzer program, which is Mascot. Unix time stamps are seconds since January, 1st 1970.<br>See header:date
**IDENTITY_THRES**	This field contains the identity threshold shown by Mascot. It is computed as follows for those who always want to know how Earth spins.<br><br>Be *m* the average value of all qmatch*i* in the summary block.<br><br>This value has to be divided by 20**p*, but p is usually the famous *p* value of 0.05. Keep this in mind for the following computation:<br><br>IDENTITY_THRESHOLD = $10 * log_{10}(m)$<br><br>**This value is shown in Mascot result presentations.**<br><br>See summary:qmatch*i*
**QUERIES**	This field contains the number of queries (series of measurement) contained in the input file.<br>See header:queries
**COMMENT**	This field contains the comment associated with the experiment. This is the content of Mascot's TITLE entry. If this field isn't set or bound to the empty string, the COM field is used.<br>An empty string is possible for this variable.<br>See parameters:TITLE and parameters:COM
**CHARGE**	This field contains the content of the charge search field of Mascot.<br>This field is not the charge Mascot actually uses. In fact, Mascot ignores this field if the experiment provides a value. See here for used values during evaluation.<br>An empty string is possible for this variable.<br>See parameters:CHARGE
**SEG**	This field contains the content of the protein mass search field of Mascot.<br>This field changes all possible results significantly. Every non-empty value should be treated as a sign that this computation has been done for experimental reason. **Never ever use results of this input file in a comparison of/groups with other results.**<br>An empty string is possible and expected for this variable.<br>See parameters:SEG
**CUTOUT**	This field contains the cutout of the parent mass.<br>An empty string is possible and expected for this variable.<br>See parameters:CUTOUT
**USERID**	This field contains the user ID.<br>An empty string is possible and expected for this variable.<br>See parameters:USERID

Code O, An input file ending line

This line is given once for each occurence of the *Code I* input file description line. A severe error can be considered if this line doesn't appear after a *Code I* line or before the second occurrence of that line. A roleback is suggested for the last data set.

The number directly following the **O** will match the number following the **I** in the corresponding input file description line.

Fields of the input file ending line

name	content
**SUCCESS**	Code that indicates either success by a value of 1 or a failure in case of a value of 0. In the later case it is advisable to consider a roleback.<br>Note that one failure in a containing query results in a failure of the input file. Nothing is said about other query results. They may be usable.

Code B, The Beginning of a new query processing

This line introduces the beginning of a new query processing. At least one query usually is part of an input file. All following lines up to EOF (in case of en error) or a Code E-line are related to this individual file.

A query is characterised by a list of ions representing a peaklist with some additional informations. Most of these informations are extracted by programs out of the raw data file of the mass spectrometer.

Fields of the beginning of a new query processing

name	content
**QUERY**	This is the number of the query (1-based) in the current input file. The number doesn't need to be consecutive.<br>This number can be used for direct references into the source file. The numbering is identical.
**CHARGE**	This is the charge of the precursor found in the current query.<br>See summary:qexp*i*'s second value<br>There exists a relation between **CHARGE**, **MASS** and **PRECURSOR**, see here.
**MASS**	This is the uncharged mass of the precursor molecule.<br>See summary:qmass*i*<br>There exists a relation between **CHARGE**, **MASS** and **PRECURSOR**, see here.
**PRECURSOR**	This is the value of the famous value of *m/z* of the charged precursor ion.<br>See summary:qexp*i*'s first value<br>There exists a relation between **CHARGE**, **MASS** and **PRECURSOR**, with **H** being the mass of a Hydrogen (either monoisotopic or average depending on **AVERAGE**!) it is:<br><br>**MASS** = **PRECURSOR** * **CHARGE** - **H** * **CHARGE**

**MATCH**	This field contains the number of matching peptides at different sites of different proteins with their mass matching the range spanned by the **PEPTIDE_TOL** around the **MASS** value.<br>See summary:qmatch*i*
**IDENTITY_THRES**	This field contains the identity threshold. It is computed as follows from **MATCH** known as the MOWSE score threshold (MOWSE = More Of Weird Statistical Errors).<br><br>**IDENTITY_THRES** = 10 * log_{10}(**MATCH**)<br><br>Note that this value isn't shown by Mascot usually. Mascot uses the overall value for the complete file explained here.<br><br>See summary:qmatch*i*
**HOMOLOGY_THRES**	This field contains the homology threshold computed by Mascot. The homology theshold is shown by Mascot in its overviews as threshold of *significant homology* with $p < 0.05$ **if this value is less than IDENTITY_THRES**.<br>The author suggests max(IDENTITY_THRES, HOMOLOGY_THRES) currently as a good threshold of convincing results.<br>See summary:qplughole*i*
**TITLE**	This field contains a string describing the title of the peak serie.<br>An empty string is possible for this variable.<br>See query*i*:title
**PEAKLIST**	This field contains the list of peaks measured by the instrument. Each peak is a couple of value and intensity (in this order) delimited by a colon. The peaks itself are delimited by commas.<br>See query*i*:Ions1
**STRCHARGE**	This is the charge of the precursor determined by mascot.<br>See query*i*:charge=

Code E, The Ending of a query processing

This line is given once for each occurence of the *Code B* beginning of a new query processing. A severe error can be considered if this line doesn't appear after a *Code B* line or before the second occurrence of that line. A roleback is strongly suggested for the last data set.

The number directly following the **E** will match the number following the **B** of the beginning of the new query processing.

Fields of the ending of a query processing

name	content
**SUCCESS**	Code that indicates either success by a value of 1 or a failure in case of a value of 0. In the later case it is strongly suggested to do a data roleback.

Code P, A Protein data line

This line shows data of the summary section relating a distinct query.

Some lines in the summary section may be invalidated, which is normal, because the summary section contains protein choices of Mascot for the "best hit". This doesn't contain all different peak lists if more than one peak list is given at all. Thus, the HITNUMBER may have non-consecutive numbers if more than one query is used in an input file.

Fields of a protein data line

name	content
**PROTEIN**	This is the name of the protein Mascot assigned to a specific hit. The kind and specification of the name is database depending.<br>A string containing a comma is possible for this variable.<br>The **PROTEIN** field with the **QUERY** field should be unique in one input file.<br>See summary:h*i*'s first element
**HITNUMBER**	This is the number under which the **PROTEIN** is positioned in the hit list.<br>The smaller the number, the better the hit of the protein.<br>The **HITNUMBER** field with the **QUERY** field are unique in one input file.<br>See the *i* in summary:h*i*
**TOTAL_SCORE**	This is the total score of the proteine. It is the result of a complexe formula known by Matrix Science. In general, is is the sum of each individual peptide in the input file that matches this protein. Even low scored peptides contribute their score to the sum, maybe partially.<br><br>One of the things not mentioned very well in the documentation is the fact, that even **different** peptides generated by **one** peak list will add their amount of score to the total score.<br>This is the reason why even with only one peak list in the input file the protein hit list and the peptide hit list differ.<br><br>See summary:h*i*'s second element
**TOTAL_MASS**	This is the computed mass of the protein.<br>See summary:h*i*'s forth element
**MISSED_CLEAVAGE**	This is the number of missed cleavages detected by Mascot for the **PEPTIDE**.<br>See summary:h*i*_q*j*'s first element
**QUERY**	This is the *j* in summary:h*i*_q*j* and is equal to the **QUERY** of a Code B line.
**PEPTIDE**	This field contains the modified peptide sequence. Every ambiguous amino acid code (B, X, Z) has been replaced by a valid amino acid code. Every variable modification is annotated by a modification code. It isn't impossible that even the termini are modificated. Exactly in this case the modifications of the termini is delimited by a period from the peptide's sequence.<br>An example is "@.HMIIM~KKM" which has two modifications, one at the N-terminus, one other at the M in the middle.

	See summary:h*i*_q*j*'s seventh element
**`PEPTIDE_MASS`**	This is the computed mass of the peptide without charge.<br>See summary:h*i*_q*j*'s second element
**`PEPTIDE_START`**	This is the position of the peptide in the protein (1-based).<br>See summary:h*i*_q*j*'s forth element
**`PEPTIDE_SCORE`**	This is the score of the **`PEPTIDE`** Mascot has computed.<br>The value is more or less useful depending on the thresholds.<br>See summary:h*i*_q*j*'s tenth element
**`OCCURANCES`**	This is the number of occurances of the **`PEPTIDE`**'s mass in the pool of the masses of each possible peptide in the protein. The information may be useful for PMF searches.<br>See summary:h*i*_q*j*'s eleventh element
**`MATCHING_FRAGMENTS`**	This is number of matching ions.<br>We still need to know which ions are counted both as "found" and which ion series are possible.<br>See summary:h*i*_q*j*'s sixth element
**`MATCHING_PEAKS`**	This is number of matching peaks in the list of peaks for this peptide.<br>See summary:h*i*_q*j*'s eighth element
**`SERIES_FOUND`**	This is a list of ion series found in the peak list matching the theoretical spektrum of the peptide.<br><br>This string should have 17 characters (which is known to be different in some Mascot versions) being either `0` (not found), `1` (more than a random peak), `2` (scored peak).

Elements of the **`SERIES_FOUND`** string

**position**	**serie**
1	a
2	reserved, should be zero
3	a++
4	b
5	reserved, should be zero
6	b++
7	y
8	reserved, should be zero
9	y++
10	c

11	c++
12	x
13	x++
14	z
15	z++
16	z+H
17	z++H++

See summary:h*i*_q*j*'s twelveth element

**SERIES_FOUND_STR**	This is a list of ion series found in the peak list matching the theoretical spektrum of the peptide in a user readable form.<br><br>This value is the representation of **SERIES_FOUND**. Only known series are displayed with at least more than random matches. Unscored values are displayed in parentheses, scored values are displayed directly. The entries are comma-separated.<br>Example: **SERIES_FOUND**=`"00010020000000000"` leads to **SERIES_FOUND_STR**=`"(b),y"`<br>See summary:h*i*_q*j*'s twelveth element

Code F, A peptide data line

This line shows data of the peptides section relating a distinct query. AG Sickmann of RVZ uses this data preferable.

The **HITNUMBER** field with the **PROTEIN_NUMBER** field and the **QUERY** field are unique in one input file.

Fields of a peptide data line

name	content
**PROTEIN**	This is the name of the protein Mascot assigned to a specific hit. The kind and specification of the name is database depending.<br>A string containing a comma is possible for this variable.<br>See peptides:q*i*_p*j*'s twelfth element
**PROTEIN_NUMBER**	This is the running number of the various proteins in the list of matching protein list for a particular **PEPTIDE**.<br>The **HITNUMBER** field with the **PROTEIN_NUMBER** field and the **QUERY** field are unique in one input file.<br>See peptides:q*i*_p*j*'s twelfth element
**HITNUMBER**	This is the number under which the **PEPTIDE** is positioned in the hit list. The smaller the number, the better the hit of the peptide for one particular query.<br>The **HITNUMBER** field with the **PROTEIN_NUMBER** field and the **QUERY** field are unique in one input file.

	See the *j* in peptides:q*i*_p*j*
`TOTAL_MASS`	This is the computed mass of the protein.<br>**This field may not be set due to Mascot#s format. The value is 0.0 in this case.**<br>The value is extracted out of the summary section or the proteins section.
`MISSED_CLEAVAGE`	This is the number of missed cleavages detected by Mascot for the `PEPTIDE`.<br>See peptides:q*i*_p*j*'s first element
`QUERY`	This is the *i* in peptides:q*i*_p*j* and is equal to the `QUERY` of a Code B line.
`PEPTIDE`	This field contains the modified peptide sequence. Every ambiguous amino acid code (B, X, Z) has been replaced by a valid amino acid code. Every variable modification is annotated by a modification code. It isn't impossible that even the termini are modificated. Exactly in this case the modifications of the termini is delimited by a period from the peptide's sequence.<br>An example is "@.HMIIM~KKM" which has two modifications, one at the N-terminus, one other at the M in the middle.<br>See peptides:q*i*_p*j*'s fifth element
`PEPTIDE_MASS`	This is the computed mass of the peptide without charge.<br>See summary:h*i*_q*j*'s second element
`PEPTIDE_START`	This is the position of the peptide in the protein (1-based).<br>See peptides:q*i*_p*j*'s twelfth element
`PEPTIDE_SCORE`	This is the score of the `PEPTIDE` Mascot has computed.<br>The value is more or less useful depending on the thresholds.<br>See peptides:q*i*_p*j*'s eighth element
`OCCURANCES`	This is the number of occurances of the `PEPTIDE`'s mass in the pool of the masses of each possible peptide in the protein. The information may be useful for PMF searches.<br>See peptides:q*i*_p*j*'s twelfth element
`MATCHING_FRAGMENTS`	This is number of matching ions.<br>We still need to know which ions are counted both as "found" and which ion series are possible.<br>See peptides:q*i*_p*j*'s forth element
`MATCHING_PEAKS`	This is number of matching peaks in the list of peaks for this peptide.<br>See peptides:q*i*_p*j*'s sixth element
`SERIES_FOUND`	This is a list of ion series found in the peak list matching the theoretical spektrum of the peptide.<br><br>This string should have 17 characters (which is known to be different in some Mascot versions) being either `0` (not found), `1` (more than a random peak), `2` (scored peak).<br><br>Elements of the `SERIES_FOUND` string

<table>
<tr><td></td><td>
<table>
<tr><th>position</th><th>serie</th></tr>
<tr><td>1</td><td>a</td></tr>
<tr><td>2</td><td>reserved, should be zero</td></tr>
<tr><td>3</td><td>a++</td></tr>
<tr><td>4</td><td>b</td></tr>
<tr><td>5</td><td>reserved, should be zero</td></tr>
<tr><td>6</td><td>b++</td></tr>
<tr><td>7</td><td>y</td></tr>
<tr><td>8</td><td>reserved, should be zero</td></tr>
<tr><td>9</td><td>y++</td></tr>
<tr><td>10</td><td>c</td></tr>
<tr><td>11</td><td>c++</td></tr>
<tr><td>12</td><td>x</td></tr>
<tr><td>13</td><td>x++</td></tr>
<tr><td>14</td><td>z</td></tr>
<tr><td>15</td><td>z++</td></tr>
<tr><td>16</td><td>z+H</td></tr>
<tr><td>17</td><td>z++H++</td></tr>
</table>
See peptides:qi_pj's nineth element
</td></tr>
<tr><td><code>SERIES_FOUND_STR</code></td><td>This is a list of ion series found in the peak list matching the theoretical spektrum of the peptide in a user readable form.

This value is the representation of <code>SERIES_FOUND</code>. Only known series are displayed with at least more than random matches. Unscored values are displayed in parentheses, scored values are displayed directly. The entries are comma-separated.
Example: <code>SERIES_FOUND</code><code>="00010020000000000"</code> leads to <code>SERIES_FOUND_STR</code><code>="(b),y"</code>
See peptides:qi_pj's nineth element</td></tr>
<tr><td><code>N-TERM_AA_STR</code></td><td>This is a list of n-terminal amino acids found for this peptide sequence.

One character per amino acid.
- if ending peptide.
The i'th chacter of <code>N-TERM_AA_STR</code> corresponds to the i'th of <code>C-TERM_AA_STR</code>.
See peptides:qi_pj_terms</td></tr>
</table>

**C-TERM_AA_STR**	This is a list of c-terminal amino acids found for this peptide sequence.<br><br>One character per amino acid.<br>- if ending peptide.<br>The i'th chacter of N-TERM_AA_STR corresponds to the i'th of C-TERM_AA_STR.<br>See peptides:q*i*_p*j*_terms
